# Effect of Coffee Consumption on Non-Alcoholic Fatty Liver Disease Incidence, Prevalence and Risk of Significant Liver Fibrosis: Systematic Review with Meta-Analysis of Observational Studies

**DOI:** 10.3390/nu13093042

**Published:** 2021-08-30

**Authors:** Maryam Ebadi, Stephen Ip, Rahima A. Bhanji, Aldo J. Montano-Loza

**Affiliations:** Division of Gastroenterology & Liver Unit, University of Alberta Hospital, Edmonton, AB T6G 2X8, Canada; sip@ualberta.ca (S.I.); rbhanji@ualberta.ca (R.A.B.)

**Keywords:** coffee, caffeine, NAFLD, liver fibrosis

## Abstract

Background and aim: Non-alcoholic fatty liver disease (NAFLD) is the most common cause of chronic liver disease worldwide. Given the anti-fibrotic and antioxidant properties of coffee, this systematic review and meta-analysis aims to provide updated results on the impact of coffee consumption on NAFLD incidence, prevalence, and risk of significant liver fibrosis. Methods: We conducted a comprehensive search in MEDLINE (OvidSP) and Scopus from January 2010 through January 2021. Relative risks for the highest versus the lowest level of coffee consumption were pooled using random-effects models. Heterogeneity and publication bias were evaluated using the Higgins’ *I*^2^ statistic and Egger’s regression test, respectively. Results: Eleven articles consisting of two case-control studies, eight cross-sectional studies, and one prospective cohort study were included in the meta-analysis. Of those, three studies with 92,075 subjects were included in the analysis for NAFLD incidence, eight studies with 9558 subjects for NAFLD prevalence, and five with 4303 subjects were used for the analysis of liver fibrosis. There was no association between coffee consumption and NAFLD incidence (RR 0.88, 95% CI 0.63–1.25, *p =* 0.48) or NAFLD prevalence (RR 0.88, 95% CI 0.76–1.02, *p* = 0.09). The meta-analysis showed coffee consumption to be significantly associated with a 35% decreased odds of significant liver fibrosis (RR 0.65, 95% CI 0.54–0.78, *p <* 0.00001). There was no heterogeneity (*I*^2^ = 11%, *p* = 0.34) and no evidence of publication bias (*p* = 0.134). Conclusion: This meta-analysis supports the protective role of coffee consumption on significant liver fibrosis in patients with NAFLD. However, the threshold of coffee consumption to achieve hepatoprotective effects needs to be established in prospective trials.

## 1. Introduction

Non-alcoholic fatty liver disease (NAFLD) is the most common cause of chronic liver disease in North America and is one of the main health complications seen around the world. Recently, a change in its nomenclature to Metabolic-Associated Fatty Liver Disease (MAFLD) [1,2] has been proposed by a group of experts. This acronym seems to better capture pathogenesis but has not been widely accepted. In this review, we will continue to use NAFLD.

The estimated prevalence of NAFLD is 25–30% in the adult population [3] and it is a leading indication for liver transplantation [4]. NAFLD constitutes a histological spectrum starting with lipid accumulation in hepatocytes (simple steatosis) without significant inflammation or liver fibrosis, with the potential to progress to non-alcoholic steatohepatitis (NASH) (varying stages of fibrosis) and subsequently to cirrhosis development with an increased risk of decompensation and hepatocellular carcinoma (HCC) [5]. Worsening fibrosis is the main factor associated with liver-related morbidity and mortality in patients with NAFLD [6]. NAFLD is frequently diagnosed as an incidental finding based on abnormal serum liver function tests and “bright liver” on imaging or hepatomegaly [7,8] in subjects without significant alcohol consumption and with no other cause of chronic liver disease. 

Genetic and lifestyle factors play an important role in the pathogenesis of NAFLD. Thus, current management focuses mainly on lifestyle adaptations to reduce steatosis and risk of fibrosis development. Numerous studies have shown beneficial effects of coffee consumption in various diseases including diabetes mellitus type 2, cardiovascular disease, HCC, and NAFLD [9,10,11]. The anti-fibrotic and cytoprotective antioxidant properties of coffee may inhibit onset of hepatic steatosis or progression to steatohepatitis with fibrosis [12,13]. Natural polyphenols, present in coffee, have been shown to protect the liver from fibrosis, which is a major surrogate for negative clinical outcomes in NASH [14]. In experimental studies, decaffeinated coffee attenuated liver steatosis, oxidative stress, and inflammation in rats on a high-fat diet [15]. However, coffee consumption has no impact on the serum levels of an inflammatory mediator, leptin, in patients with mild to severe hepatic steatosis [16]. 

Nevertheless, the effects of coffee consumption on NAFLD risk have been conflicting. Therefore, this systematic review and meta-analysis aims to provide updated results on the impact of coffee consumption on NAFLD incidence, prevalence, and liver fibrosis risk.

## 2. Material & Methods

### 2.1. Study Search and Selection

We conducted a comprehensive search for titles, abstracts, and keywords in MEDLINE (OvidSP) and Scopus using the search term: (coffee OR caffeine) combined with (NASH OR NAFL* OR “nonalcoholic fatty*” OR “nonalcoholic steatohepatitis” OR “liver steatosis” OR “hepatic steatosis” OR “steatohep*” OR “hepatic fibrosis” OR “liver fibrosis” OR “hepatosteatosis”) from January 2010 through January 2021. Reference lists of included manuscripts and published meta-analyses were also checked manually. The search was limited to human studies written in English.

The inclusion criteria were as follows: (a) case-control, cross-sectional, or cohort studies; (b) published as original studies to evaluate the impact of coffee consumption on NAFLD incidence, prevalence, and risk of significant liver fibrosis; (c) including relative risks (RRs), odds ratios (ORs), or hazard ratios (HRs) with 95% confidence intervals (95% CIs) or adequate data to calculate them; (d) NAFLD diagnosis by serum biomarkers of liver injury or imaging studies (i.e., ultrasound, computed tomography, etc.), elastography, or liver histological evaluation.

We excluded studies without sufficient statistics or adequate data for risk estimates, lack of biochemical tests and imaging to evaluate liver injury, no description of coffee intake measurement (e.g., questionnaire, dietary recall, food record), use of coffee derivatives, no discrimination between coffee or caffeine as the main exposure, or if NAFLD incidence, prevalence, or significant liver fibrosis was not the main outcome of interest. Studies evaluating the association between coffee consumption and NAFLD in the general population or in patients with NAFLD are included. We consider significant liver fibrosis as a histological score greater than or equal to F2 according METAVIR or elastography >8 kPa. All these studies assessed liver fibrosis in a single time-point and therefore, we present the results as liver fibrosis associated with NAFLD but not the fibrosis progression in NAFLD, as assessing progression requires more than one-time measurements.

### 2.2. Data Extraction

For each study, the author names and year of publication, data on study design, population, NAFLD diagnosis, coffee consumption assessment, steatosis and fibrosis severity classification, RRs/HRs/ORs estimates for extreme categories of coffee intake and corresponding 95% CIs, the adjustments for confounding variables, and main findings were extracted. We did not communicate with the study authors regarding missing information. The methodological quality of each article was assessed using the Newcastle−Ottawa Scale (NOS) [17], which assesses each study in three areas including the selection of participants and measurement of exposure, the comparability of study groups, and evaluation of outcome. The adapted Newcastle–Ottawa scale [18], giving a maximum of five stars for selection, two stars for comparability, and three stars for outcome, was applied for cross-sectional studies. Assessment of study eligibility and quality was conducted independently by ME and AML, and any discrepancies regarding the inclusion/exclusion of studies or data extraction were determined by a consensus of the reviewers.

### 2.3. Statistical Analysis

In this meta-analysis, ORs of the case-control and the cross-sectional studies as well as HRs of the cohort study were used as an estimate of RRs. RRs for the highest versus the lowest level of coffee consumption were pooled using random-effects models. If more than one RR estimate was reported, the one most comprehensively adjusted for potential confounding factors was used. For studies reporting only the number of cases and controls for each category of coffee consumption, crude RRs and the corresponding 95% CIs were computed from these frequencies. Studies with risk estimates of two categories (e.g., coffee drinkers versus non-drinkers) were also integrated. 

Heterogeneity was evaluated using the Higgins’ *I*^2^ statistic with values ≤25%, ≤50%, ≤75%, and >75% representing no, small, moderate, and significant heterogeneity, respectively [19]. We also examined the stability of the results by performing a sensitivity analysis, in which we calculated pooled RRs and 95% CIs after excluding one study at a time from the analysis. Potential publication bias was assessed using Egger’s regression test [20] with the evidence of small-study effects on P_Egger_ < 0.1. All analyses were performed using Stata 16.0 (StataCorp, https://www.stata.com) and Review Manager (Rev-Man) version 5.4 with *p*-values < 0.05 considered as statistically significant.

## 3. Results

### 3.1. Publication Selection and Quality Assessment

**Figure 1:**
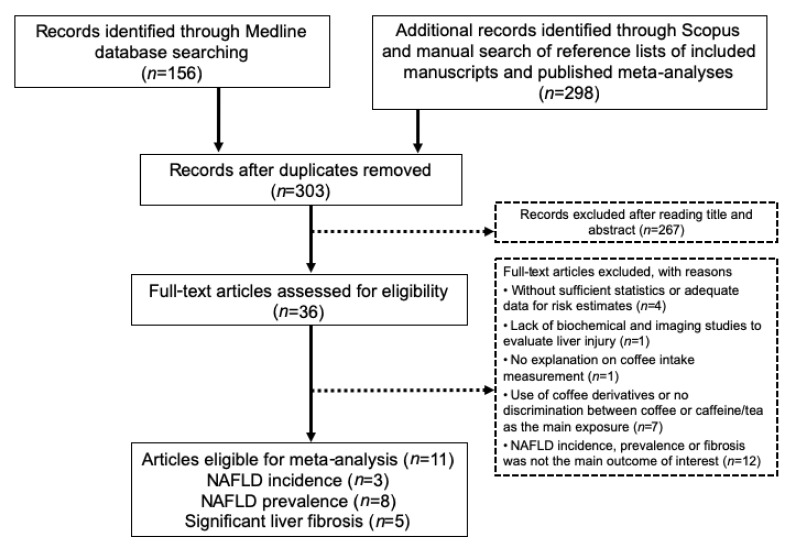
Flow chart of literature search and inclusion of studies in the meta-analysis. Abbreviation: NAFLD, Non-alcoholic fatty liver disease.

The initial search strategy yielded 303 potentially relevant articles after the exclusion of duplicates. Two hundred and sixty-seven articles were excluded after screening titles and abstracts, leaving the remaining 36 articles for a full-text article review. According to inclusion and exclusion criteria, 11 articles fulfilled the eligibility criteria and, therefore, were included in the data analysis [11,16,21,22,23,24,25,26,27,28,29]. The detailed flow chart of the selection of eligible literature included in the meta-analysis is presented in Figure 1. Included studies are presented in Table 1. These 11 articles consist of two case-control studies, eight cross-sectional studies, and one prospective cohort study. From these studies, three studies with 92,075 subjects [21,22,23] were included in the analysis for NAFLD incidence, eight studies with 9558 subjects for NAFLD prevalence [11,16,23,24,25,26,27,28], and five studies with 4303 subjects were used for the analysis of significant liver fibrosis risk [11,23,28,29].

Data on coffee consumption was collected by interviewer-administrated questionnaire in three studies [23,25,29]. The severity of hepatic steatosis was identified using liver ultrasonography in six studies [16,23,24,25,26,27] while elastography [28] and biopsy [11] were performed in two studies. Fibrosis severity was determined based on liver histology in two studies [11,29]. Four articles had a score of seven stars [16,22,27,29], six with eight stars [11,21,23,24,26,28], and only one article was nine stars [25] by NOS. Reporting items for systematic reviews and meta-analysis are included as Supplementary Materials (Supplementary Table S1). 

### 3.2. Coffee Consumption and NAFLD

Association between coffee consumption and NAFLD incidence, prevalence, and liver fibrosis risk was investigated in previous observational studies. In a cross-sectional study of 347 subjects, an inverse association was reported between coffee consumption (3 cups/day) and degree of liver fibrosis, but there was no association with NAFLD prevalence. Similarly, when a subgroup of subjects without fatty liver at baseline (*n* = 147) was prospectively followed for 7 years, no association was found between coffee intake and NAFLD onset assessed by liver ultrasound (OR 0.72, 95% CI 0.28–1.85; *p* = 0.50). Although poorer lifestyles were noticed in subjects with high coffee intake (three cups/day), controlling for lifestyle factors (smoking, diet, and physical activity) did not reveal any association with NAFLD onset [23]. No association was found between the amount of coffee consumption at baseline and NAFLD incidence in a cohort of 91,436 subjects who were followed for a mean of 2.8 years; however, an increase in the amount of coffee consumption, rather than the absolute amount, was associated with reduced NAFLD incidence (HR 0.94, 95% CI 0.90–0.98) [21].

A recent cross-sectional study in 555 NAFLD patients found no association between coffee drinking and severe hepatic steatosis, defined as CAP 280 dB/m. Coffee consumption was associated with a lower risk of advanced fibrosis (defined as liver stiffness measurement (LSM) ≥10 kPa) after adjusting for confounders (OR 0.49, 95% CI 0.27–0.88; *p* = 0.02) [28]. Similarly, an Italian study demonstrated that coffee drinking may not be protective against the severity of hepatic steatosis. In a cross-sectional study of middle-aged Italian participants, higher coffee intake was not associated with a lower risk of NAFLD, or severity of liver steatosis when adjusted for age, sex, smoking status, presence of diabetes, cancer, acute myocardial infarction, waist circumference, blood pressure, daily energy, and alcohol intake [24]. 

Increased intake of coffee, rather than total caffeine intake from other sources, was protective against the progression of fibrosis in patients with NASH. In a cross-sectional study of biopsy-proven NASH patients and ultrasound-negative controls, coffee intake was higher in controls and patients with NASH stage 0–1 compared to those with stage 2–4 fibrosis, suggesting the beneficial effects of coffee in preventing fibrosis development [9]. The metabolic background of patients might be an important determinant of coffee effect on liver fibrosis, as an inverse association between coffee intake and advanced fibrosis was only observed among patients with NAFLD and low insulin resistance (HOMA-IR <4.3), with no protective effect in patients with higher HOMA-IR [11].

A dose-dependent protective effect of coffee on the prevalence [16] and severity of hepatic steatosis [30] was reported in previous studies. More than three cups of coffee per day was associated with a lower risk of NAFLD when compared to intake of <2 cups per day [31]. 

### 3.3. Meta-Analysis 

#### 3.3.1. Coffee Consumption and NAFLD Incidence

**Figure 2:**
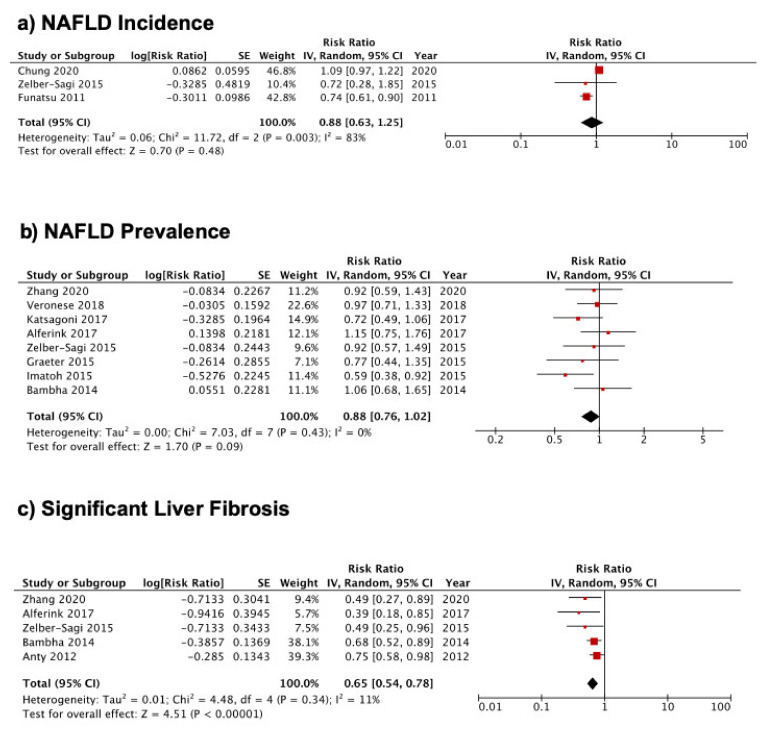
Forest plot for the association between coffee consumption and (**a**) NAFLD incidence, (**b**) NAFLD prevalence, and (**c**) significant liver fibrosis. Relative risk for the highest versus the lowest level of coffee consumption was pooled using random-effects models.

During the three to seven years’ follow up, no association was seen between coffee consumption and NAFLD incidence in subjects without NAFLD at baseline (RR 0.88, 95% CI 0.63–1.25, *p* = 0.48; Figure 2a). There was significant heterogeneity (*I*^2^ = 83%, *p* = 0.003) but no evidence of publication bias (*p* = 0.674). In a sensitivity analysis that excluded the study by Chung et al. [21], a reduction in heterogeneity (*I*^2^ = 0%, *p* = 0.96) was associated with a significant reduction in the incidence of NAFLD (RR 0.74, 95% CI 0.61, 0.89, *p* = 0.002). Due to the small number of studies, we were unable to conduct subgroup analyses to determine the sources of heterogeneity. However, NAFLD has a prolonged natural course, and variable follow-up times between studies ranging from 2.8 years in the study by Chung et al. [21] to 7 years in a study by Zelber-Sagi [23] may explain some of the heterogeneity. 

#### 3.3.2. Coffee Consumption and NAFLD Prevalence

A total of one case-control [27] and seven cross-sectional [11,16,23,24,25,26,28] studies were pooled to evaluate the association between coffee consumption and NAFLD prevalence. The analysis revealed no significant association between coffee consumption and NAFLD prevalence (RR 0.88, 95% CI 0.76–1.02, *p* = 0.09; Figure 2b). There was no evidence of heterogeneity (*I*^2^ = 0%, *p* = 0.43) or publication bias (*p* = 0.699). To check the effect of individual studies on the pooled RR, a sensitivity analysis was performed with exclusion of one trial [25] (Alferink et al. 2017), resulting in a significant reduction in the risk of NAFLD prevalence by coffee consumption (RR 0.85, 95% CI 0.72–0.99, *p* = 0.04). There was no significant change in RR or *I^2^* values with removal of other studies. Subgroup analyses according to the study design, questionnaire administration, study quality, and energy intake adjustment revealed that the association was significant in studies with lower quality, without adjustment for total energy intake, or when the self-reported questionnaire was applied (Table 2a).

#### 3.3.3. Coffee Consumption and Risk of Significant Liver Fibrosis

Meta-analysis with a randomized-effect model including five studies showed that coffee consumption was significantly associated with 35% decreased odds of liver fibrosis (RR 0.65, 95% CI 0.54–0.78, *p* < 0.00001; Figure 2c) with no heterogeneity (*I*^2^ = 11%, *p* = 0.34). Egger’s linear regression test was performed and showed no publication bias (*p* = 0.134). Sensitivity analysis by excluding one study at a time revealed high stability of the results and no single influential study on the pooled RR, which varied from 0.59 (95% CI 0.43–0.80) when the study from Bambha et al. [11] was excluded to 0.68 (95% CI 0.57–0.80) when the study from Alferink et al. [25] was removed. Results did not change after performing a subgroup analysis based on study design, questionnaire administration, study quality, and energy intake adjustment (Table 2b). Results were also stable in both NAFLD patients (RR 0.64, 95% CI 0.50–0.82, *p* = 0.0004) and the general population (RR 0.60, 95% CI 0.40–0.89, *p* = 0.01).

## 4. Discussion

Association between coffee consumption and NAFLD incidence, prevalence, and significant liver fibrosis risk was quantitatively assessed in our meta-analysis. We found increased coffee consumption was significantly associated with a reduced risk of significant liver fibrosis. This association was robust and consistent between different subgroups and in our sensitivity analysis. However, no association was observed between coffee consumption and NAFLD incidence or prevalence.

In the majority of studies, liver steatosis and fibrosis were not quantified histologically and were diagnosed based on imaging studies. The sensitivity of ultrasound in revealing hepatic steatosis varies between 60 to 94% depending on the population of the interest [32], and ultrasonography can only detect steatosis with >2.5–20% liver fat content [33]. Although liver biopsy is the gold standard for NAFLD diagnosis, it has drawbacks such as invasive nature, sampling inconsistency, and low acceptance by patients and is unsuitable for longitudinal monitoring. Moreover, it may not be appropriate to perform liver biopsies in low-risk subjects; biopsy-proven NAFLD patients may not be representative of the general population with NAFLD. Non-invasive biomarkers have been suggested for the diagnosis of NASH and fibrosis [33], and biopsies are reserved for individuals with NASH diagnosis based on imaging and non-invasive assessments [34]. The use of various modalities and cut-offs to establish the severity of steatosis or fibrosis can affect the overall assessment; thus, a common method should be standardized.

Few meta-analyses were conducted to evaluate the relationship between coffee and NAFLD. In a meta-analysis of four cross-sectional studies and two case-control studies, no association was found between total caffeine consumption (mg/day) with either the prevalence or the risk of liver fibrosis related to NAFLD. However, when stratified by the type of caffeine intake, regular coffee caffeine intake (mg/day) significantly decreased the risk of liver fibrosis or development of NAFLD [35]. Lower risk and prevalence of NAFLD in coffee drinkers and the protective effect of coffee on liver fibrosis have also been reported in other studies [36,37,38]. In comparison to these meta-analyses, we included a larger number and more recent studies in the analysis. In contrast to prior studies, we specifically investigated the association between coffee consumption and NAFLD incidence. We also conducted a subgroup analysis by study design, questionnaire administration, study quality, energy intake adjustment, and population of interest which has not been previously performed. A systematic review by Sewter et al. included studies assessing the progression of NAFLD to hepatic fibrosis and/or cirrhosis in relation to coffee consumption [38]; however, our study aimed to conduct a meta-analysis to determine the impact of coffee consumption on NAFLD incidence, prevalence, and fibrosis risk in the general population or patients with NAFLD. Contrary to the systematic review by Sewter et al., studies without detailed information for risk estimates were excluded from our meta-analysis. 

Some limitations should be noted in interpreting the results of the current meta-analysis. First, this analysis was limited to the English language and exclusion of manuscripts in other languages or unpublished studies might contribute to publication bias. The majority of the studies in this area were cross-sectional studies showing an association but not able to determine causality and limiting the capability to evaluate chronological associations. Reverse causality is a common concern in cross-sectional studies as the predictor and outcome are assessed at the same time. The risk of bias and the quality of the studies were assessed using the NOS, a commonly used tool for observational studies. Although a conventional sampling method was applied in most of the studies, comparison between responders and non-responders was not reported, contributing to self-selection bias. 

Observational studies are limited by the inability to account for confounding factors, including nutritional and lifestyle behaviors (diet, physical activity, smoking, obesity, insulin resistance, and body fat distribution). Several comorbidities affecting the severity of NAFLD such as diabetes and obesity differ between coffee drinkers and non-drinkers, and a lack of adjustment can lead to bias in the assessment of the effect of the exposure. Daily energy intake may be higher in subjects who regularly drink coffee with additives such as sugar and cream [39], and without adjusting for daily calorie intake, it is impossible to properly establish the association between coffee intake and NAFLD. Prospective studies should evaluate dietary habits and medications that might impact liver fibrosis such as thiazolidinediones, statins, vitamin E, and omega 3 as potential confounders [40]. In this meta-analysis, the association between coffee consumption and liver fibrosis remained significant in studies adjusting for calorie intake and other confounders such as age, sex, BMI, HOMA-IR, and physical activity.

One of the main challenges in these studies is to properly assess an individual patient’s coffee consumption. Coffee intake was evaluated using a questionnaire at a single time-point and therefore the impact of changes in habitual coffee intake was not determined. The validity of the questionnaire and blinded interview are also important considerations. Retrospective self-reported questionnaires infer the risk of recall or reporting bias [41]. Illustrative exemplification of standard drinks/coffee cups is another approach to mitigate error in estimating drink size [42] which needs to be considered in future studies. Classification of regular coffee intake differed between studies, and the unit was not standardized across included studies. Therefore, the optimal dose that correlates with the greatest reduced risk would need to be established in future clinical trials.

Potential beneficial components of regular coffee need to be determined in prospective studies as coffee contains several compounds including caffeine, polyphenol (chlorogenic acid), and diterpenes (cafestol and kahweol) [43]. Experimental studies show coffee mitigates hepatic fibrosis by down-regulating profibrogenic genes [44,45], inhibiting adhesion and activation of hepatic stellate cells [12], and activating nuclear factor erythroid 2-related factor 2, inducing the antioxidant enzymes system [45]. It was also suggested that coffee attenuates hepatic steatosis through upregulation of β-oxidation, downregulation of de novo lipogenesis, suppression of oxidative stress [46], and prevention of adipogenic hepatic steatosis [47]. The antioxidant properties of caffeine [48] and the inhibitory effect of kahweol on hepatic inflammation [49] may help to attenuate the development of NASH (Supplementary Figure S1). A recent meta-analysis of randomized clinical trials suggests the anti-inflammatory impact of coffee might be related to its ability to lower tumor necrosis factor alfa levels. There was no significant association with other markers including interleukin −6 and C-reactive protein in this meta-analysis [50].

## 5. Conclusions

This meta-analysis confirms the protective role of regular coffee consumption on the risk of significant liver fibrosis. No association was observed between coffee consumption and NAFLD incidence or prevalence. In the absence of approved drug therapy, coffee should be recommended in conjunction with lifestyle changes. The threshold of coffee consumption to achieve its protective effect needs to be established in prospective trials. It is still unclear which element of coffee confers benefit. Prospective studies with a comprehensive evaluation of coffee intake over time will be important to clarify the amount, type, preparation method, and pattern of coffee consumption that has the most benefit in liver disease. The impact of possible confounding factors such as enhanced nutritional status or physical activity cannot be excluded.

## Figures and Tables

**Table 1:** Summary of studies investigating the impact of coffee consumption on NAFLD incidence, prevalence, and liver fibrosis.

Study	Study Design	Population	NAFLD Diagnosis	Steatosis Severity	Fibrosis Severity	Coffee Intake Measurement	Main Finding	RR/HR/OR Estimates for Extreme Categories of Coffee Intake (95% CI)	Adjustments for Confounding Variables	NOS
Chung et al., 2020 [21]	Retrospective longitudinal cohort	91,436 subjects participating in a comprehensive health-screening program were followed for a mean of 2.8 years, 13,362 (15% developed fatty liver)	Ultrasound	Steatosis incidence: increase in liver echogenicity compared with the renal cortex echogenicity		Self-administered food frequency questionnaire	No association between fatty liver incidence and the amount of coffee consumption at baseline	HR 1.09 (0.97–1.22)	Age, sex, education, exercise, smoking, alcohol intake, centre and year, BMI, total energy intake, triglyceride, LDL-C, HDL-C, glucose, alanine aminotransferase, aspartate aminotransferase, change of alcohol, change of BMI, and change of exercise	8
Zhang et al., 2020 [28]	Cross-sectional	555 NAFLD patients from multi-centre hepatology clinics	Radiological features, liver histology, or elevated alanine aminotransferase or aspartate aminotransferase levels with a CAP value ≥ 248 dB/m	Elastography: severe hepatic steatosis: CAP threshold of 280 dB/m	Elastography: advanced fibrosis: LSM threshold of 10 kPa	Standardized, self-administered questionnaires	Inverse association between coffee consumption and advanced fibrosis No association between coffee consumption and severe hepatic steatosis	OR 0.49 (0.27–0.88), *p* = 0.02 OR 0.92 (0.59–1.44), *p* = 0.71	Age, sex, smoking and alcohol status, coffee, tea, and soft drinks drinker, time spent on different physical activities, energy expenditure, physical activity level and meeting physical activity guidelines (%), obesity and type 2 diabetes, and participating centre	8
Veronese et al., 2018 [24]	Cross-sectional	2819 randomly sampled participants from electoral rolls Absence of fatty liver in 1627 subjects and 916 with NAFLD (134 patients with Liver Steatosis Score of 6)	Ultrasound	Fatty liver score ranged from 0 to 6, with higher values indicating a greater severity		Self-reported validated semi-quantitative food frequency questionnaire	No association between coffee consumption and lower odds of liver steatosis	OR 0.97 (0.71–1.32), *p* = 0.84	Age, sex, smoking status, presence of diabetes, gastric ulcer, cancer, acute myocardial infarction, waist circumference, systolic and diastolic blood pressure, daily energy, and alcohol intake	8
Alferink et al., 2017 [25]	Cross-sectional	Population-based cohort of 2424 participants (35% steatosis)	Ultrasound	Presence or absence of a hyper-echogenic liver parenchyma	Transient elastography: liver stiffness measurements (LSM) ≥8.0 kPa	Validated 389-item food frequency questionnaire	Independent association between frequent coffee consumption and lower probability of significant liver fibrosis No significant association between coffee intake and steatosis	OR 0.39 (0.18–0.86), *p* = 0.005 OR 1.15 (0.75–1.77), *p* = 0.192	Tea, energy intake, BMI, gender, age, steatosis, alanine aminotransferase, excessive alcohol intake, current or former smoking and HOMA-IR, soda consumption, cream and sugar use, dietary quality, and physical activity Tea, energy intake, BMI, gender, age, HOMA-IR, excessive alcohol intake, current or former smoking	9
Katsagoni et al., 2017 [27]	Case-control	100 newly ultrasound-proven NAFLD patients (21 with NASH), 55 healthy controls matched for age, sex, and BMI	Elevated alanine aminotransferase and/or gamma- glutamyl transpeptidase levels, elastography, and evidence of hepatic steatosis on ultrasound (available for 85) and/or compatible liver histology (*n* = 32)	Evidence of hepatic steatosis at ultrasonography Biopsy: NASH Diagnosis: NAFLD Activity score (NAS) ≥ 5		Semi-quantitative validated food frequency questionnaire	Inverse association between coffee intake and NAFLD presence (OR 0.68, 95% CI 0.49–0.94, *p* = 0.02) Lost its significance once adjusted for adiponectin and TNF-a	OR 0.72 (0.49–1.04), *p* = 0.07	Age, sex, waist circumference, HOMA-IR, adiponectin, and TNF-a	7
Zelber-Sagi et al., 2015 [23]	Cross-sectional and prospective cohort	Cross-sectional cohort 347 general population, 31% diagnosed with NAFLD Prospective cohort A subgroup of patients without fatty liver at baseline who were followed up for 7 years (*n* = 147)	Ultrasound and SteatoTest	Hepatorenal index (HRI) on US, NashTest (borderline NASH or definite NASH) SteatoTest (≥5%, ≥S1–S2)	FibroTest (significant fibrosis (≥F2))	Interviewer-administrated questionnaire and detailed semiquantitative food-frequency questionnaire	Cross-sectional cohort: Inverse association between coffee consumption and significant liver fibrosis and no association with the development of steatosis Prospective cohort: No association between coffee consumption and NAFLD incidence	NAFLD prevalence 0.92 (0.57–1.50), *p* = 0.75 Hepatic Fibrosis:0.49 (0.25–0.97), *p* = 0.04 NAFLD Incidence: 0.72 (0.28–1.85), *p* = 0.501	Current smoking, sugar intake, physical activity (minutes per week), serum cholesterol levels, and dietary fat and calories intake Current smoking, sugar intake, physical activity	8
Imatoh et al., 2015 [16]	Cross-sectional	1024 Japanese male workers receiving annual health checkups Non-steatosis (*n* = 270) Steatosis (*n* = 754)	Ultrasound	No, mild, or moderate-to-severe hepatic steatosis		Self-reported questionnaire	Dose-dependent protective effect of coffee on the prevalence of hepatic steatosis	OR 0.59 (0.38–0.90), *p* = 0.03	BMI, age, smoking status, alcohol drinking, and green tea consumption	7
Graeter et al., 2015 [26]	Cross-sectional	Random population-based sample with 1452 subjects (381 diagnosed with hepatic steatosis)	Ultrasound	No steatosis and steatosis grade I, II and III		Standardized questionnaire	No association between hepatic steatosis and coffee consumption	OR 0.77 (0.44–1.34), *p* = 0.81	Age, BMI, gender, metabolic syndrome, and physical activity	8
Bambha et al., 2014 [11]	Cross-sectional	782 biopsy-proven NAFLD patients; Advanced fibrosis (>stage 2) in 25% (*n* = 199) NASH (definite or probable): in 79% (*n* = 616)	Biopsy	Presence versus absence of NASH histology ((1) definite steatohepatitis; (2) definitely not steatohepatitis; and (3) borderline steatohepatitis)	None to moderate (≤Stage 2) or advanced (>Stage 2)	Self-reported validated dietary questionnaire	Significant association between coffee intake and decreased odds of advanced fibrosis in patients with lower HOMA-IR	OR 0.68 (0.52–0.89), *p* = 0.005	Age, sex, race, waist circumference, aspartate transaminase, gamma-glutamyl transferase diabetes, smoking, alcohol, biopsy length, HOMA-IR, and interaction between coffee and HOMA-IR	8
Anty et al., 2012 [29]	Cross-sectional	195 severely and morbidly obese patients, referred for bariatric surgery of which NASH was present in 19.5%	Biopsy	NAFLD activity score (NAS) simple steatosis (NAS ≤ 2), borderline (3≤ NAS ≤ 4), or definitive NASH (NAS ≥ 5)	Significant fibrosis (F ≥ 2)	Interviewer- administered questionnaire	Regular coffee consumption was an independent protective factor for significant fibrosis	OR: 0.752 (0.578–0.980), *p* = 0.04	Aspartate aminotransferase, presence of NASH, presence of the metabolic syndrome, and level of HOMA-IR	7
Funatsu et al., 2011 [22]	Nested case-control	1236 subjects followed for 5 years; of those, 164 males with fatty liver were matched (age, BMI, and exercise level) with 328 without fatty liver	Ultrasound	Steatosis incidence: a bright liver, an increase in the liver−kidney contrast, and/or a decrease in liver deep echo		Self-administered questionnaire	Daily coffee intake was inversely associated with fatty liver development	0.74 (0.61–0.89), *p* = 0.001	Age, BMI, exercise, daily alcohol intake, and changes in BMI, exercise level, and daily alcohol intake over time	7

Abbreviation: BMI, body mass index; HR, hazard ratio; NAFLD, non-alcoholic fatty liver disease; NASH, alcoholic steatohepatitis; NOS, the Newcastle−Ottawa Scale; OR, odds ratio; RR, relative risk.

**Table 2:** Subgroup meta-analysis of coffee consumption and (a) NAFLD prevalence, (b) significant liver fibrosis stratified by study design, questionnaire administration, study quality, and energy intake adjustment.

Subgroup	Number of Studies	Pooled RR	Heterogeneity
NAFLD Prevalence			
All studies	8	0.88 (0.76–1.02), *p* = 0.09	*I*^2^ = 0%, *p* = 0.43
*Study design* Case-control Cross-sectional	1 7	0.72 (0.49–1.06), *p* = 0.09 0.91(0.77–1.07), *p* = 0.25	*I*^2^ = 0%, *p* = 0.44
*Questionnaire* Self-reported Interviewed	6 2	0.84 (0.71–0.99), *p* = 0.04 1.04 (0.76–1.43), *p* = 0.80	*I*^2^ = 3%, *p* = 0.40 *I*^2^ = 0%, *p* = 0.50
*NOS score* 7 8-9	2 6	0.66 (0.49–0.88), *p* = 0.005 0.97 (0.82–1.16), *p* = 0.76	*I*^2^ = 3%, *p* = 0.50 *I*^2^ = 0%, *p* = 0.91
*Energy Intake Adjustment*			
Yes No	2 6	1.03 (0.80–1.32), *p* = 0.82 0.81 (0.67–0.97), *p* = 0.02	*I*^2^ = 0%, *p* = 0.53 *I*^2^ = 0%, *p* = 0.50
Significant Liver Fibrosis			
All studies	5	0.65 (0.54–0.78), *p* < 0.00001	*I*^2^ = 11%, *p* = 0.34
*Questionnaire* Self-reported Interviewed	2 3	0.64 (0.50–0.82), *p* = 0.0004 0.60 (0.40–0.89), *p* = 0.01	*I*^2^ = 0%, *p* = 0.33 *I*^2^ = 42%, *p* = 0.18
*NOS score* 7 8-9	1 4	0.75 (0.58–0.98), *p* = 0.03 0.60 (0.48–0.75), *p* < 0.00001	*I*^2^ = 0%, *p* = 0.42
*Energy Intake Adjustment*			
Yes No	2 3	0.44 (0.27–0.74), *p* = 0.002 0.69 (0.58–0.83), *p* < 0.0001	*I*^2^ = 0%, *p* = 0.66 *I*^2^ = 0%, *p* = 0.43
*Population* NAFLD patients General population	2 3	0.64 (0.50–0.82), *p* = 0.0004 0.60 (0.40–0.89), *p* = 0.01	*I*^2^ = 0%, *p* = 0.33 *I*^2^ = 42%, *p* = 0.18

Abbreviation: NAFLD, non-alcoholic fatty liver disease; NOS, the Newcastle−Ottawa Scale; RR, relative risk.

## Data Availability

The data presented in this study are available in Table 1 and Table 2.

## Supplementary Materials

The following are available online at https://www.mdpi.com/article/10.3390/nu13093042/s1, Figure S1: Mechanisms by which coffee impacts hepatic steatosis and fibrosis; Table S1: PRISMA (Preferred Reporting Items for Systematic reviews and Meta-Analysis).
